# Intermittent High-Dose Vitamin D3 Administration in Neonates with Multiple Comorbidities and Vitamin D Insufficiency

**DOI:** 10.3390/children11030328

**Published:** 2024-03-09

**Authors:** Catalin Cirstoveanu, Iulia Ionita, Carmina Georgescu, Carmen Heriseanu, Corina Maria Vasile, Mihaela Bizubac

**Affiliations:** 1 Department of Neonatal Intensive Care, “Carol Davila” University of Medicine and Pharmacy, 020021 Bucharest, Romania; catalin.cirstoveanu@umfcd.ro (C.C.); mihaela.bizubac@drd.umfcd.ro (M.B.); 2 Neonatal Intensive Care Unit, “M.S. Curie” Children’s Hospital, Constantin Brâncoveanu Boulevard, No. 20, 4th District, 041451 Bucharest, Romania; carmina.georgescu@drd.umfcd.ro (C.G.); mariana-carmen.iliescu@drd.umfcd.ro (C.H.); 3 Faculty of Medicine, “Carol Davila” University of Medicine and Pharmacy, 020021 Bucharest, Romania; 4 Pediatric Cardiology, “M.S. Curie” Children’s Hospital, Constantin Brâncoveanu Boulevard, No. 20, 4th District, 041451 Bucharest, Romania; corina.vasile93@gmail.com; 5 Department of Pediatric and Adult Congenital Cardiology, University Hospital of Bordeaux, 33600 Pessac, France

**Keywords:** insufficiency of vitamin D, cholecalciferol, newborn, neonatal intensive care unit

## Abstract

Background: Neonates have an increased risk of vitamin D insufficiency due to the inadequate supplementation of mothers and infants after birth. Insufficiency of vitamin D is frequently detected in critically ill patients and is associated with disease severity and mortality. There is yet to be a consensus on the appropriate regimen of vitamin D3 supplementation in high-risk infants. Aim: The main objectives of this study were to determine the prevalence of vitamin D insufficiency in neonates with severe comorbidities and to evaluate whether high-dose vitamin D3 oral administration leads to normal plasmatic concentrations without side effects. Methods: The current study was a randomized, prospective trial of 150 patients admitted to the Neonatal Intensive Care Unit (NICU) at Maria Sklodowska Curie Emergency Children’s Hospital in Bucharest. Patients were divided into three subgroups based on the chronological order of their admission date. Each subgroup received a different pharmaceutical product of vitamin D3. We administered a dosage of 10,000 IU/kg of vitamin D3 orally in three steps, as follows: at admission, one week after admission, and one month from the first administration, targeting a serum 25-hydroxyvitamin D concentration of at least 40 ng/mL. Results: Most neonates (68%) achieved an optimum vitamin D level after one month, even though only 15% of patients had an optimum concentration at admission. After the first high dose of vitamin D3, there was a 27% increase in the mean vitamin D plasmatic level compared to admission levels. However, after one month, the concentrations decreased in all subgroups due to the gap of three weeks between the last two administrations. Conclusions: An intermittent, weekly high-dose vitamin D3 oral administration leads to a steadier increase and normalization of vitamin D concentration in most critically ill neonates. However, high-dose vitamin D3 administered orally after three weeks decreases vitamin D levels in this high-risk population.

## 1. Introduction

Vitamin D serves multiple immune roles by modulating the genes that encode proteins for cell proliferation, differentiation, and apoptosis, which is highly important in critically ill patients [1,2]. Cathelicidin, a peptide stimulated by vitamin D, has antimicrobial activity against bacteria, fungi, and viruses [3]. Vitamin D inhibits the production of pro-inflammatory cytokines and stimulates the production of anti-inflammatory cytokines [4]. Epidemiological studies linked vitamin D deficiency in children to a more severe course of illness, such as lower respiratory tract infections or respiratory syncytial virus bronchiolitis [2]. Furthermore, vitamin D aids bone development by increasing the intestinal absorption of calcium and phosphorus to shape the bone mineral matrix correctly [5].

Most studies found a strong link between vitamin D insufficiency and disease severity, mortality, prolonged hospitalization in ICU, higher risk of sepsis, and acute kidney injury [6,7,8]. A prospective study at Boston Children’s Hospital’s PICU found that vitamin D deficiency at admission was correlated with mediocre clinical outcomes, higher scores of illness severity, and an increased risk of requiring vasopressor support [9]. The authors concluded that these results were secondary to the multiple roles played by vitamin D in immunological regulation, inflammatory response, and calcium homeostasis.

Vitamin D3 (cholecalciferol) is produced in the skin after exposure to ultraviolet B radiation, converting 7-dehydrocholesterol to pre-vitamin D3 and vitamin D3 [10]. Sun exposure can be limited due to northern latitude, an individual’s skin color, or a lack thereof in hospitalized patients. Vitamin D3 can also be procured through supplements and certain food products [11,12].

Vitamin D deficiency is associated with many diseases, from rickets and osteomalacia to neurological and inflammatory diseases. Vitamin D deficiency during pregnancy can lead to fetal and maternal morbidity, including gestational diabetes, increased rates of cesarean section, and a small-for-gestational-age infant [13,14,15]. Infants may develop impaired neurocognitive development, reduced bone mineral content with an increased risk of fractures, skeletal deformities, and asymptomatic or symptomatic hypocalcemia, leading to cardiomyopathy, tetany, and seizures [16,17].

Despite this, the routine monitoring of vitamin D levels is uncommon, particularly for the neonatal population. Moreover, 25(OH)D is preferred for evaluating the serum concentration of vitamin D, and 1,25(OH)2D is the least favorable vitamin D serum concentration indicator due to its short half-life. Only severe vitamin D deficiency typically leads to decreased 1,25(OH)2D levels [18].

Based on the Recommended Dietary Allowance data, the National Academy of Science, U.S. considers vitamin D concentration inadequate when 25(OH)D is <20 ng/mL [19]. Meanwhile, the International Global Consensus Guidelines 2016 define vitamin D sufficiency as 25(OH)D > 50 nmol/L (>20 ng/mL), insufficiency between 30 and 50 nmol/L (12–20 ng/mL), and deficiency as <30 nmol/L (<12 ng/mL) [20].

Several conditions may cause insufficient vitamin D serum levels in infants, including intestinal malabsorption due to low cardiac output syndrome, short bowel syndrome, inflammatory bowel disease, cystic fibrosis, biliary disease, or intestinal bacterial overgrowth. Increased vitamin D catabolism can be caused by certain medications, such as anticonvulsants, glucocorticoids, antifungals, and highly active antiretroviral therapies.

Maternal circulating vitamin D concentration during pregnancy is the primary determinant of neonatal vitamin D status. Both routine screening and the administration of vitamin D3 to mothers improve vitamin D levels in newborns [21]. The Institute of Medicine U.S. recommends 400–600 IU/day vitamin D3 intake during pregnancy [22], while the Endocrine Society recommends a higher intake of 1500–2000 IU/day [23]. However, recent studies suggest that higher vitamin D3 intake (2000–4000 IU/day) leads to higher plasmatic concentrations of 25(OH)D in pregnant women, decreasing the incidence of pregnancy complications [24].

Breast milk, despite being the ideal form of infant feeding, is a suboptimal source of vitamin D. Exclusive breastfeeding may increase the risk of vitamin D insufficiency unless additional intake is provided via food, supplementation, or sufficient sunlight exposure [25]. A mother’s breast milk with adequate vitamin D levels contains approximately 22 IU/L [26], substantially lower than the recommended daily intake. When comparing the contribution of genetic factors to maternal vitamin D levels, it was observed that maternal vitamin D concentrations predict 19% of neonatal vitamin D concentration. At the same time, genetics have a minor influence [27].

Although numerous oral vitamin D3 supplements exist today, not enough studies vouch for an appropriate dose of vitamin D3 to gain a sufficient concentration in newborns or pregnant women. The American Academy of Pediatrics, the Canadian Pediatric Society, and the European Society for Pediatric Endocrinology recommend 400 IU/day of vitamin D3 supplementation for infants. However, the European Society for Pediatric Gastroenterology, Hepatology, and Nutrition recommends a higher dose of 800–1000 IU/day for high-risk infants [28]. The adequate dose of vitamin D3 supplementation in the neonatal and pediatric population and the optimal administration frequency to maintain sufficient levels in the body are still under debate.

The purpose of this study is to create a protocol to manage high-risk patients with vitamin D insufficiency and to administer the necessary doses at a specific time-frequency to quickly reach an adequate serum level of 25(OH)D.

## 2. Materials and Methods

### 2.1. Study Design and Participants

A randomized, analytical, prospective, single-center study was conducted between April 2020 and March 2023 that included 150 patients with multiple severe anomalies, such as cardiac malformations, respiratory diseases, gastrointestinal disorders, neurologic conditions, and genetic syndromes. Most of these anomalies required surgical intervention during the one-month follow-up. 

Patients were divided into three subgroups, Lot 1, Lot 2, and Lot 3, each composed of 50 patients based on their admission order. Each subgroup received a different pharmaceutical vitamin D3 product. All patients were orally administered high doses of vitamin D3 until they reached positive outcomes. All patients were monitored for at least one month after admission, and clinical data were prospectively collected from medical records. 

Additionally, 25(OH)D serum concentrations were measured in ng/mL, with the following correlation between the two units of measurement: 1 international unit (IU) of vitamin D, approximately equivalent to 0.025 micrograms. This conversion factor bridges the gap between the recommended intake and the observed serum concentrations.

In total, 26 patients (17.3%) were excluded from the study due to their intolerance to oral intake through conditions such as esophageal atresia or ileus and cases where the study protocol was not adequately implemented.

### 2.2. Vitamin D3 Administration Protocol

The study’s two main objectives were to highlight the high prevalence of vitamin D insufficiency in patients hospitalized in the Neonatal Intensive Care Unit and to show that high-dose intermittent vitamin D3 oral administration leads to the steady and balanced normalization of vitamin D concentrations in neonates with a severe general status and multiple comorbidities without side effects.

The one-month time frame for the treatment plan and follow-up corresponds to the medium hospitalization period in our unit.

**Figure 1:**
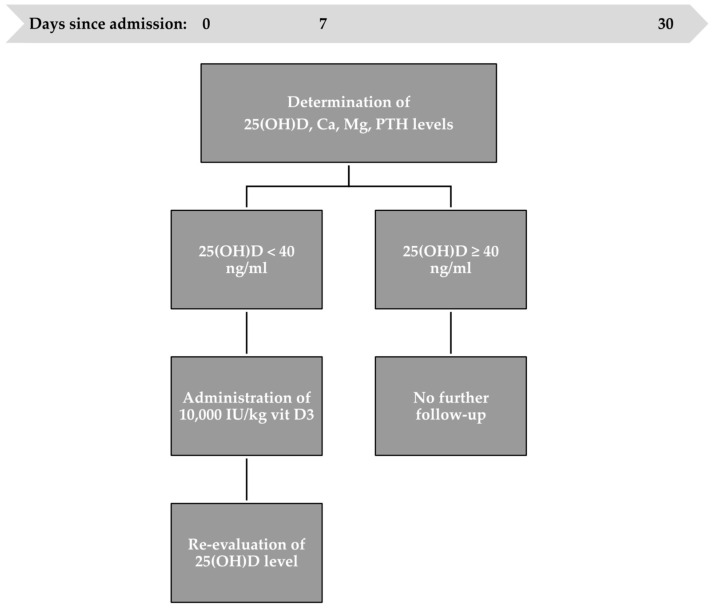
Study protocol on admission, day seven, and day thirty.

High-dose vitamin D3 supplementation was defined as 10,000 IU/kg administered orally in three steps: at admission, one week after admission, and one month after the first administration (Figure 1). None of the newborns had been exposed to sunlight nor received vitamin D3 supplementation before admission. The newborns’ sources of vitamin D3 were high doses administered orally according to the study protocol, along with intake provided by parenteral or enteral nutrition. Our unit’s parenteral nutrition protocol includes the administration of fat-soluble vitamins (Vitalipid N), containing a maximum daily dose of 400 IU of vitamin D. Patients able to be fed received breast milk or milk formula, offering 22 IU/L or 40–44 IU/100 mL of vitamin D, respectively, depending on the formula.

Vitamin D status was determined by dosing the serum concentration of 25(OH)D before and after each administration. Calcium, magnesium, and parathormone levels were determined at admission. The method used to determine 25(OH)D and parathormone levels was CLIA (chemiluminescence immunoassays). Meanwhile, the colorimetric method was used for calcium and magnesium. All tests were undertaken in our hospital’s laboratory.

A positive outcome was a concentration of 25(OH)D ≥ 40 ng/mL, while a negative result was <40 ng/mL [23]. High-dose vitamin D3 administration was interrupted after reaching an optimum level of 25(OH)D.

The first step was to measure the serum concentration of 25(OH)D upon admission. Infants with an optimum level did not receive vitamin D3 supplementation, unlike infants with an inadequate level who received a high dose of vitamin D3. The serum concentration of 25(OH)D was re-evaluated after 24 h to appreciate the therapeutic response. Follow-up was discontinued for 25(OH)D > 40 ng/mL patients.

After one week, vitamin D status was determined again to analyze the sustained effect. Patients who did not reach a minimum level of 40 ng/mL received a second high dose of vitamin D3. The 25(OH)D level was redetermined one day after the second administration.

The third step of the protocol was to evaluate vitamin D serum levels one month after the first administration. Patients who did not reach a minimum level of 40 ng/mL received a third high dose of vitamin D3. The serum concentration of 25(OH)D was re-evaluated after the last administration.

The gap between the first and second vitamin D3 oral administrations was seven days to attain an average absorption time in high-risk neonates, most of whom underwent surgical interventions in the first week of hospitalization. For the second gap, the 15-day half-life of 25(OH)D [29] was followed by administering the third and final high dose after three weeks, corresponding to the hospital’s one-month average hospitalization period. This allowed for a better evaluation of the absorption process and the necessary time for the body to ingest vitamin D3.

### 2.3. Statistical Analysis

The collected data underwent meticulous processing and comprehensive statistical analysis using various software tools. Microsoft Excel 2007 was used for the initial data organization and preparation. Subsequently, Google Docs and Google Sheets were used for further statistical interpretation. These software tools facilitated the application of the appropriate statistical methods to explore and extract meaningful information from the dataset. Descriptive statistics were used to summarize the data, while inferential statistical techniques, such as *t*-tests or ANOVA, were applied to assess the significant differences or correlations between variables. Using these software resources provided accuracy and rigor to our statistical analysis, contributing to the correctness of our research results.

### 2.4. Ethical Considerations

Approval was obtained from our Hospital Ethics Committee to ensure the ethical conduct of the study (No. 7057/20 February 2020). This institutional approval provided the necessary ethical framework for this research. 

## 3. Results

During the past three years (April 2020–March 2023), 150 critically ill neonates were included in the study (Table 1).

**Figure 2:**
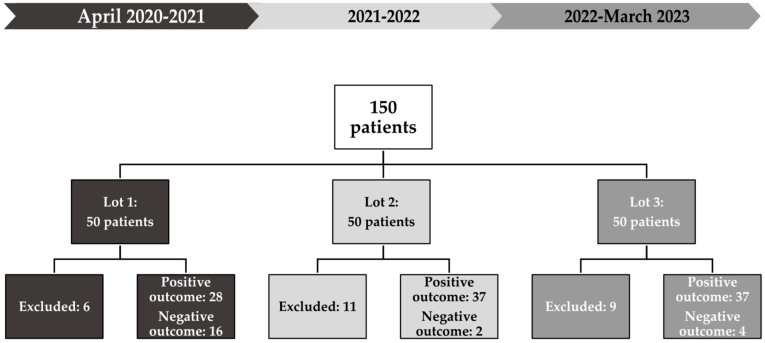
Study design and overall outcome in the three subgroups at the end of the study.

Most neonates (68%) achieved an optimum vitamin D level at the end of the month of the study. In comparison, 14.7% had persistent vitamin D insufficiency despite supplementation, and the rest (17.3%) were excluded from the study (Figure 2).

The overall mean vitamin D level on admission was 24.2 ng/mL (*p* = 0.04), with differences between the three subgroups, as follows: subgroup 1—mean level of 23.9 ng/mL, subgroup 2—20.9 ng/mL, and subgroup 3—27.9 ng/mL.

**Figure 3:**
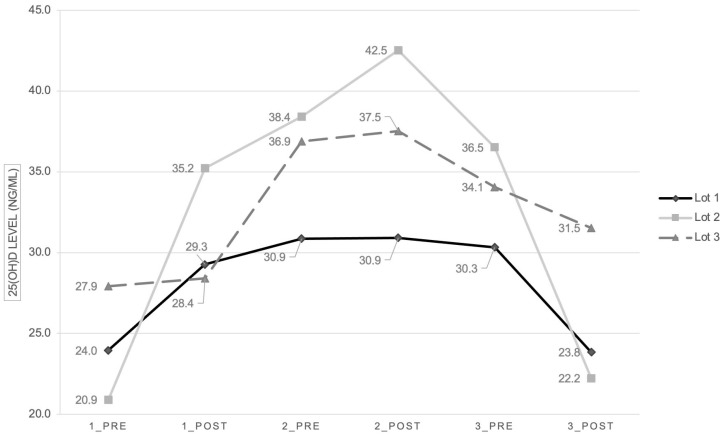
Mean 25(OH)D levels in the three subgroups at admission (1_PRE—before the first administration, 1_POST—after the first administration), after one week (2_PRE—before the second administration, 2_POST—after the second administration), and after one month since admission (3_PRE—before the third administration, 3_POST—after the third administration).

After the first administration, the vitamin D concentration increased, with the mean level increasing to 30.9 ng/mL (*p* = 0.147). The second subgroup had the most robust growth (Figure 3).

One week after the first dose administration, all the subgroups had a slight increase in vitamin D concentration (*p* = 0.176), with the highest increase in the third subgroup. After the second administration (*p* = 0.152), there was an increase only in the second and third subgroups, while the vitamin D level remained unchanged in the first group.

One month after admission and three weeks after the second administration, vitamin D levels slightly decreased (*p* = 0.673). After the third administration, the vitamin D concentration decreased even more (*p* = 0.486).

**Figure 4:**
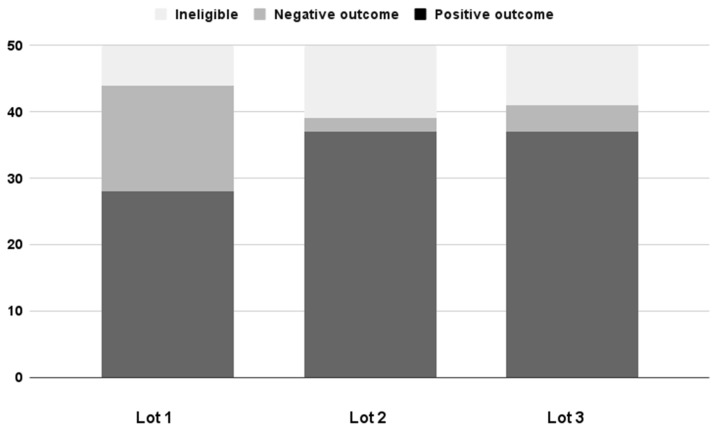
Comparison of the results obtained in the three subgroups after administering three high doses of vitamin D3 at one month following admission. Positive outcome—25(OH)D concentration ≥ 40 ng/mL; negative outcome—25(OH)D concentration < 40 ng/mL.

Lot 1 included patients with comorbidities, half of them receiving surgical interventions while hospitalized, and a quarter of patients with gastrointestinal diseases associated with impaired intestinal absorption. After one month following admission, more than half of the patients (56%) showed a positive response to high-dose vitamin D3 administration, reaching an optimum serum concentration (Figure 4). In contrast, only 25% of premature infants had a positive outcome. A third of neonates (32%) did not reach a level ≥ 40 ng/mL.

In Lot 2, a smaller percentage of patients received surgical interventions while hospitalized (34%), and almost a third of patients suffered from gastrointestinal disorders. Only 8% of patients had an optimum 25(OH)D level upon admission, while all premature infants had vitamin D insufficiency. However, only 4% did not reach an optimum vitamin D concentration, resulting in 74% of patients with positive outcomes. A higher percentage of premature babies (78.5%) reached a positive outcome.

Lot 3 had the highest percentage of infants with gastrointestinal dysfunction and surgical conditions. At admission, 26% of them had an optimum level of vitamin D. Nonetheless, 74% of patients had favorable outcomes after three administrations of high-dose vitamin D3. A similar percentage of premature infants (76.9%) attained optimum vitamin D levels.

**Figure 5:**
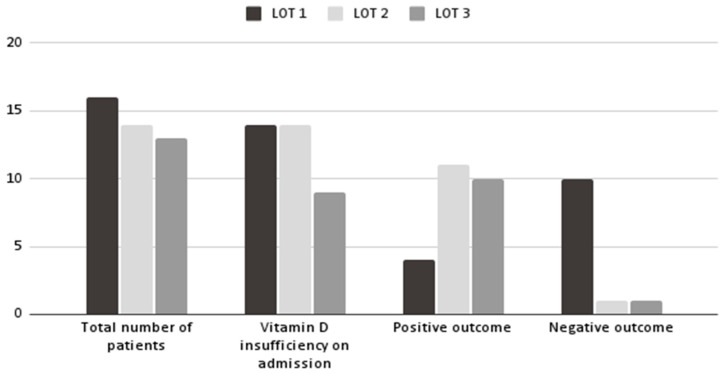
Results found in premature babies at admission and after the three high-dose vitamin D3 administrations.

In total, 43 premature babies were enrolled in the study, with 86% having an inadequate vitamin D level at admission. After the three high-dose vitamin D3 administrations, more than half of them (58.1%) achieved a positive outcome (Figure 5). 

All patients had standard serum concentrations of calcium, magnesium, and parathormone. Infants with vitamin D deficiency had a mean calcium level equal to 9.5 mg/dL in the first subgroup, 9.2 mg/dL in subgroup 2, and 9.5 mg/dL in subgroup 3. In contrast, the mean magnesium level was 2 mg/dL, 1.7 mg/dL, and 1.9 mg/dL, respectively. Neonates from subgroup 1 had mean parathormone levels of 47.1 pg/mL, subgroup 2 had a mean level of 71.7 pg/mL, and subgroup 3 had a mean level of 50.5 pg/mL. 

## 4. Discussion

Over the three-year timeline of the study, which overlapped with the COVID-19 pandemic, only 15.5% of patients had an optimum vitamin D level on admission. This indicates an extremely high prevalence of vitamin D insufficiency (84.4%) in the Neonatal Intensive Care Unit. Results were similar regarding premature babies who had a prevalence of vitamin D insufficiency of 86%. Other studies also found a high prevalence of vitamin D insufficiency in patients admitted to neonatal and pediatric intensive care units, ranging from 50% to 90% [30].

A higher percentage of patients from the third Lot (24%) had an optimum level of 25(OH)D on admission, compared to 14.5% in the first and only 8% in the second Lot. We hypothesize that this difference in vitamin D insufficiency prevalence can be explained by the fact that infants in Lot 3 were born in 2022–2023, towards the end of the COVID-19 pandemic, which led to increased awareness regarding vitamin D supplementation. The publicity revolving around the multiple immunomodulatory roles of vitamin D led to an increased supplementation in pregnant women and a better serum concentration in newborns [31]. 

Pre-term infants have an increased risk of vitamin D insufficiency compared to full-term infants [32]. Moreover, a low maternal vitamin D concentration represents a significant risk factor for premature birth [21]. The current study found a similarly high prevalence of vitamin D insufficiency in both pre-term and full-term neonates (86% vs. 83.8%). However, a smaller percentage of pre-term infants reached a normalization of 25(OH)D concentration at the end of the month of the study, compared to full-term infants (58.1% vs. 71.9%). The correlation between low gestational age and vitamin D insufficiency may be correlated with often delayed enteral feeding, intolerance to oral intake, and a high risk of necrotizing enterocolitis, which can lead to short bowel syndrome and intestinal malabsorption [33]. The aforementioned highlights the importance of monitoring and supplementing vitamin D in premature infants [34].

Most studies found no association between vitamin D concentration and the cause of hospitalization, nor between medical and surgical patients [35,36]. However, Rippel et al. found a double prevalence of vitamin D insufficiency in cardiac versus non-cardiac patients (40% vs. 22%) [37]. Half of the patients included in our study had surgical conditions, with the highest percentage represented by congenital heart diseases, gastrointestinal malformations, or necrotizing enterocolitis. All these conditions are associated with variable degrees of intestinal malabsorption, increasing the risk of vitamin D insufficiency [38]. 

After closely monitoring critically ill infants, we observed that the standard doses outlined in former protocols were not providing adequate serum concentrations of vitamin D. Therefore, the aim was to adjust our approach to ensure that infants receive the optimum care and treatment required to achieve optimal health outcomes, keeping in mind the high-risk population admitted in the NICU.

The recommended daily dose of vitamin D3 supplementation in Romania for children up to 18 months of age is between 400 and 800 IU/day. However, in some cases, particularly in the neonatal population, there is a need for an alternative approach to the daily low-dose vitamin D3 regimen due to poor adherence [39]. Furthermore, for neonates requiring surgical intervention, the time before surgery does not allow for the 2 to 3 months of low-dose vitamin D3 intake needed to achieve the optimum serum concentration of 25(OH)D [40].

Most international guidelines recommend low-dose daily oral supplementation with vitamin D3 for all infants. A systematic review regarding the administration of high-dose vitamin D3 in pediatric patients showed that enteral loading of 10,000 IU/kg (maximum 400,000 IU) is the most appropriate regimen for the rapid and safe normalization of vitamin D status [41]. The oral administration of a high dose leads to rapid absorption into the circulation, rapid liver hydroxylation, and a peak of 25(OH)D within a few days [42]. In the current study, three intermittent high-dose vitamin D3 oral administrations led to an optimum 25(OH)D level in 68% of patients. High-dose oral administration was associated with a gradual elevation of the serum concentration rather than a rapid increase. Huynh et al. proved that a single bolus delivery of 50,000 IU achieved higher 25(OH)D repletion rates after two weeks compared with daily dosing of 400 IU, even though serum concentrations were similar at 3–4 months of age [43]. Thus, periodic administration of a high dose of vitamin D3 is easier, more efficient, and safer, especially in newborns with severe comorbidities.

When comparing the efficacy of weekly high-dose oral supplementation with the same high-dose administered at an interval of three weeks, we observed a significant difference in efficacy. Weekly administration led to a steady increase in vitamin D plasmatic concentration until reaching a plateau. However, there was a decrease in the serum level of 25(OH)D when the gap between high-dose oral administrations was three weeks. The high-dose vitamin D3 administered at a three-week gap (10,000 IU/kg/dose) was equivalent to a higher-than-recommended daily dose of oral vitamin D3 (476 IU/kg/day). This further proves that low-dose oral supplementation is insufficient in high-risk infants. Therefore, new guidelines for vitamin D supplementation are needed for this category of patients [44].

There was a difference in the efficacy of vitamin D supplementation between the three lots. The most robust growth of vitamin D level (68.4%) was in Lot 2 (from a mean level of 20.9 ng/mL to 35.2 ng/mL), while Lot 1 had an increase of 21.9% (from a mean level of 24 ng/mL to 29.2 ng/mL). Lot 3 had an increase of only 1.7% (from a mean level of 27.9 ng/mL to 28.4 ng/mL). We hypothesize that this difference can be explained by the use of different pharmaceutical products of vitamin D3, which are not bioequivalent. This raises an essential question regarding the efficacy of various products used for vitamin supplementation and makes vitamin D monitoring mandatory in all infants to certify the effectiveness of vitamin supplementation.

In some hospitals, neonates are routinely initiated on low-dose cholecalciferol daily, while in others, they are screened for 25(OH)D insufficiency before initiating supplementation. However, most units have no screening or treatment protocol [45]. Numerous studies, including ours, prove that severely ill neonates and infants should undergo monitoring of vitamin D levels upon admission to correct the deficit adequately. We intend to implement a screening protocol for vitamin D insufficiency and prevention for pregnant women and newborns in Romania.

The International Global Consensus Guidelines 2016 define vitamin D toxicity as hypercalcemia and serum 25(OH)D > 250 nmol/L (100 ng/mL), leading to hypercalciuria, suppressed PTH, and symptoms such as lethargy, abdominal pain, constipation, and polyuria. In the current study, only one patient reached a high concentration of vitamin D > 100 ng/mL, with no side effects and with normal renal function tests at discharge. Moreover, this patient’s serum concentrations of calcium, magnesium, and parathormone remained within the normal range for their age. Despite public concern, vitamin D toxicity is rare and generally occurs due to genetic susceptibility [46]. For example, a higher vitamin D intake should be avoided in infants with Williams syndrome and congenital heart disease [47]. Otherwise, healthy children only develop renal adverse effects after cumulative vitamin D intake >600,000 IU, while high doses of 100,000–150,000 IU do not lead to hypercalciuria [42]. 

## 5. Limitations

The study’s primary limitation is the high number of patients excluded due to the inability of oral intake because of their pathology or incorrect administration or determination of 25(OH)D levels. Furthermore, the correlation between maternal and neonatal vitamin D levels was not evaluated due to a lack of access to the mothers’ serum vitamin D concentration, with all newborns being transferred from other hospitals nationwide. The current results could not be compared with the prevalence of vitamin D deficiency in healthy newborns in Romania due to the lack of reported data. 

## 6. Conclusions

Vitamin D deficiency is a significant burden to the neonatal population with severe comorbidities admitted in the Neonatal Intensive Care Unit. Even though some newborns may initially display normal vitamin D levels at birth due to appropriate maternal nutrition, their subsequent need for supplementation surpasses the current international guidelines. This increased requirement becomes evident shortly, particularly in infants with multiple comorbidities.

Weekly oral administration of high doses of vitamin D3 to high-risk infants is safe and efficient. A single high dose of vitamin D3 is insufficient, but multiple high weekly doses lead to a steady increase in plasmatic concentrations.

Premature infants are vulnerable to vitamin D insufficiency, increasing their risk of developing bone and immune system problems. Therefore, it is crucial to closely monitor and supplement their vitamin D levels to achieve optimal health outcomes. Taking proactive measures to address this issue can help prevent long-term complications in premature infants.

Considering these outcomes, especially concerning patients with concurrent comorbidities and extended hospitalizations, it is crucial to establish updated individualized guidelines for the neonatal population. Our study advocates for a minimum daily recommended dosage of 1000 IU/kg or an alternative weekly oral administration of 10,000 IU/kg. Such supplementation emerges as an essential requirement for high-risk patients.

## Figures and Tables

**Table 1:** Demographics and hospitalization characteristics.

		LOT 1	LOT 2	LOT 3	Overall Patients
Gender	Female	28 (56%)	18 (36%)	21 (42%)	67 (44.6%)
	Male	22 (44%)	32 (64%)	29 (58%)	83 (55.3%)
Gestational age	Term	34 (68%)	36 (72%)	37 (74%)	107 (71.3%)
	Pre-term	16 (32%)	14 (28%)	13 (26%)	43 (28.6%)
Associated comorbidities	Cardiac malformation	21 (42%)	25 (50%)	20 (40%)	66 (44%)
	Respiratory Disease	42 (84%)	39 (78%)	46 (92%)	127 (84.6%)
	Gastrointestinal malformation	11 (22%)	12 (24%)	17 (34%)	40 (26.6%)
	Necrotizing enterocolitis	2 (4%)	3 (6%)	2 (4%)	7 (4.6%)
	Tumor	3 (6%)	2 (4%)	3 (6%)	8 (5.3%)
	Neurologic disorder	12 (24%)	18 (36%)	13 (26%)	43 (28.6%)
	Renal Disease	7 (14%)	8 (16%)	8 (16%)	23 (15.3%)
	Surgical condition	29 (58%)	17 (34%)	33 (66%)	79 (52.6%)

## Data Availability

The data presented in this study are available on request from the corresponding author.
